# Diversity of Heparan Sulfate and HSV Entry: Basic Understanding and Treatment Strategies

**DOI:** 10.3390/molecules20022707

**Published:** 2015-02-05

**Authors:** Vaibhav Tiwari, Morgan S. Tarbutton, Deepak Shukla

**Affiliations:** 1 Department of Microbiology & Immunology, Chicago College of Osteopathic Medicine, Midwestern University, Downers Grove, IL 60515, USA; E-Mail: mtarbutton23@gmail.com; 2 Department of Ophthalmology and Visual Sciences, University of Illinois at Chicago, Chicago, IL 60612, USA; E-Mail: dshukla@uic.edu; 3 Department of Microbiology & Immunology, College of Medicine, University of Illinois at Chicago, Chicago, IL 60612, USA

**Keywords:** herpes simplex virus, 3-*O* sulfated heparan sulfate, 3-*O* sulfotransferase, viral entry

## Abstract

A modified form of heparan sulfate (HS) known as 3-*O*-sulfated heparan sulfate (3-*O*S HS) generates fusion receptor for herpes simplex virus (HSV) entry and spread. Primary cultures of corneal fibroblasts derived from human eye donors have shown the clinical significance of this receptor during HSV corneal infection. 3-*O*S HS- is a product of a rare enzymatic modification at C3 position of glucosamine residue which is catalyzed by 3-*O*-sulfotransferases (3-*O*STs) enzymes. From humans to zebrafish, the 3-*O*ST enzymes are highly conserved and widely expressed in cells and tissues. There are multiple forms of 3-*O*STs each producing unique subset of sulfated HS making it chemically diverse and heterogeneous. HSV infection of cells or zebrafish can be used as a unique tool to understand the structural-functional activities of HS and 3-*O*S HS and likewise, the infection can be used as a functional assay to screen phage display libraries for identifying HS and 3-*O*S HS binding peptides or small molecule inhibitors. Using this approach over 200 unique 12-mer HS and 3-*O*S HS recognizing peptides were isolated and characterized against HSV corneal infection where 3-*O*S HS is known to be a key receptor. In this review we discuss emerging role of 3-*O*S HS based therapeutic strategies in preventing viral infection and tissue damage.

## 1. Introduction

**Figure 1:**
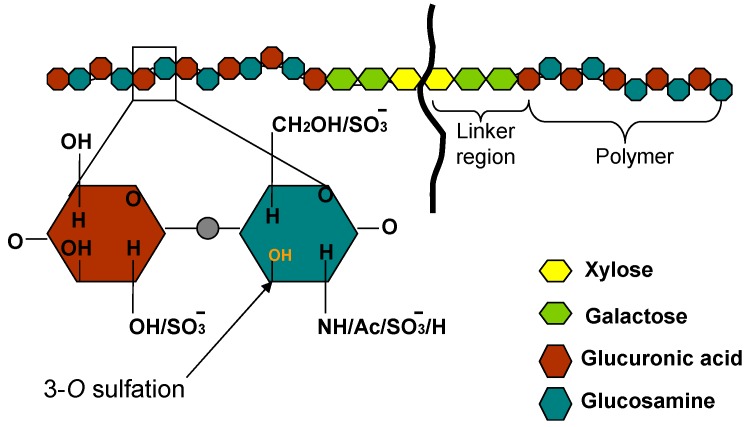
Structural features of heparan sulfate (HS). HS is a linear polymers composed of repeating uronic acid [D-glucuronic acid (GlcA) or L-iduronic acid (IdoA)] and D-glucosamine (GlcN) disaccharide subunits. Synthesized chain of HS is representing assembly of the tetrasaccharide linker region (GlcA-Gal-Gal- Xyl) at reducing end on serine residues of the protein core followed by the addition of alternating GlcA and GlcNAc residues. The chain extension is also accompanied by a series of modifications which includes 6-*O*, 3-*O* sulfations on glucosamine residue and the 2-*O* sulfation on glucuronic acid. The arrow shows the 3-*O* position of the glucosamine residue where sulfation is essential for HSV-1 glycoprotein D (gD) binding.

Heparan sulfate (HS) glycosaminoglycans are hybrid molecules with unbranched polysaccharide polymers covalently attached to the protein core [1,2,3]. The backbone of HS polymer is assembled by sequential addition of d-glucuronic acid; GlcA (or iduronic acid) alternating with N-acetylglucosamine (GlcNAc), reaching up to 100–150 residues in length (Figure 1).

The synthesized chains are then modified heterogeneously, and in domains, by multiple enzymes [4,5]. Most common among these modifications is the addition of sulfate groups at various positions within the chain, which leads to the generation of specific motifs, making HS highly attractive for microbial adherence [6,7,8]. This structural diversity which is usually concentrated in the area of sulfation enables specific binding sites for >400 proteins, including cell adhesion molecules, growth factors, chemokines, and factors regulating angiogenesis and blood coagulation [9,10,11,12]. Because of the later properties HS plays important role in multiple pathological processes such as angiogenesis, and inflammation. Heparan sulfate proteoglycans (HSPG) have also been implicated in pathogenesis induced by human herpesviruses and multiple clinically relevant viruses [6,7,13,14,15,16,17]. The abundant expression and ubiquitous presence of HS on mammalian cell surfaces makes it ideal platform to capture the viruses and wide variety of pathogens including parasites [7]. Several lines of evidence have helped define the role of HS during viral infection. For instance, multiple envelope glycoproteins and capsids from non-enveloped viruses bind to cell surface HS [17,18,19,20,21,22,23]. Further, enzymatic removal of HS by heparinase action significantly reduces viral attachment and entry [24,25,26]. Similarly cell defective in HS biosynthesis show reduced viral entry even in presence of viral entry receptor [6,27,28,29]. Also, a prior treatment of virus with soluble HS or HS-mimetic competes for cell surface HS, thereby reducing viral binding and entry [30,31,32,33,34,35,36]. Interestingly, presence of HS on spermatozoa plays a key role in the capture of human immunodeficiency virus (HIV) and its transmission to dendritic, macrophage, and T cells [37]. Similarly HS dependent uptake of HIV in brain endothelial cells aids the virus to cross the blood brain barrier [14]. In case of human papillomavirus (HPV), it has been demonstrated that HSPG play a key role in the activation of immune responses, which is critical for both vaccine development and viral pathogenesis [38]. Beside providing random docking sites to incoming virions, a special type of HS known as 3-*O*-sulfated heparan sulfate (3-*O*S HS) aids in HSV-1 penetration into host cells [6,39,40,41,42]. 3-*O*S HS is produced after a rare enzymatic modification in HS catalyzed by 3-*O*-sulfotransferases (3-*O*STs) (Figure 1) [4,5]. It has been shown that the presence of 3-*O*S HS alone makes cell susceptible to HSV infection [39]. As per current model of HSV entry, the initial attachment or binding step requires viral glycoprotein B (gB) and C (gC) binding to unmodified HS [43,44]. In the next step, a third viral glycoprotein D, (gD) specifically recognizes 3-*O*S HS, and this interaction can facilitate fusion pore formation during viral entry [45]. Various types of sulfation in HS chain are known to play critical role in viral entry, virus trafficking, and replication Table 1. For instance, 3-*O*S HS also plays a role in hepatitis B virus replication [46], while 6-*O* in HS chain potentially supports entry of cytomegalovirus [47].

**Table 1:** Role of HS modifying enzyme during viral infections.

HS Modifying Enzymes	Viral Infections	References
* H 3-*O*-Sulfotransferases-2, -3, -4, -5, -6	Herpes simplex virus infection	[6,39,41]
* ZF 3-*O*-Sulfotransferases-2, -3, -4, -5, -6	Herpes simplex virus infection	[48,49,50]
3-*O*-Sulfotransferase-1	Herpetic infection of the eye	[51]
3-*O*-Sulfotransferase-1	Hepatitis B replication	[46]
6-*O*-Sulfotransferase	Cytomegalovirus infection	[47]
6-*O*-Sulfotransferase	Coxsackievirus B3 internalization	[52]
6-*O*-Sulfotransferase	Baculovirus binding and entry	[53]
6-*O*-Sulfotransferase	Hepatitis C virus tropism	[54]
2-*O*-Sulfotransferase	Human immunodeficiency virus entry	[55]

* H: human; * ZF: Zebrafish.

**Figure 2:**
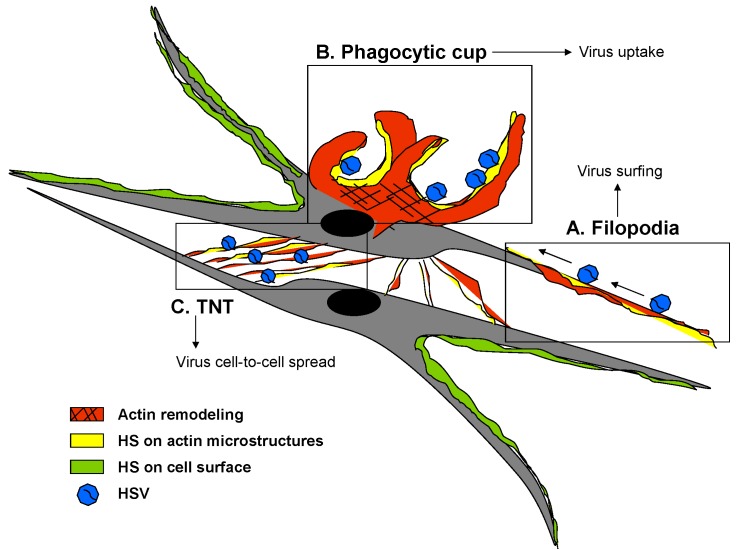
Highlights of HS involvement during novel phagocytic uptake of herpes simplex virus (HSV-1) in primary cultures of corneal fibroblasts (CF). A cartoon illustrates the expression of HS (yellow) on actin polymerized (red) regions of a HSV infected cell **A**. HS mediated virus surfing on CF-microstructures or filopodia guide the virions to reach receptor expressing cell body **B**; A novel phagocytic-uptake pathway engulfs virions (blue) via actin polymerized filopodial protrusion expressing HS **C**; Similarly networks of actin-HS rich microstructures or tunneling nanotube (TNT) between the cells help virions to spread.

Interestingly, 2-*O* sulfation in HIV is recognized by HIV glycoprotein gp120 during viral entry [14,55]. In addition, 6-*O* sulfated HS mediates coxsackievirus B3 internalization [52]. Similarly, the role of 6-*O* sulfated syndecan-1 during baculovirus binding and entry was shown recently [53]. In addition, the *O*-sulfate group of heparin is central to its inhibition of HIV [56], pseudorabies virus [57], HSV [58], and murine leukemia virus infection [59]. Conversely, *N*-sulfation of heparin is required for inhibition of respiratory syncytial virus (RSV) infection [60]. Our recent findings using HSV-1 as a model system has shown that the virus exploits HS very early during virus-cell interactions [61]. For instance HS present on filopodia microstructures guide the virus to surf and reach the cell body (Figure 2, panel A). At the cell body the virus interacts with 3-*O*S HS receptors on actin rich filopodia for phagocytic like uptake (Figure 2, panel B). The role of HS in viral surfing may be more significant than previously suggested [61]. A much deeper question remains unanswered whether surfing mechanism may also be used by the virus during cell-to cell spread via fine microstructures (Figure 2, panel C). Similarly, the question whether the surfing is guided via a specific HS-detecting radar system built-in the virus, helping it to find a suitable target cell remains unclear. Along the same lines, a phagocytic uptake model of HSV in primary cultures of corneal fibroblasts (CF) derived from human corneal donors provides a classic example of a poorly understood virus high jacking mechanism exploiting HS and the actin cytoskeleton of the host cells (Figure 2). 

## 2. Structural Diversity of HS and Implications in Corneal HSV Infection

**Figure 3:**
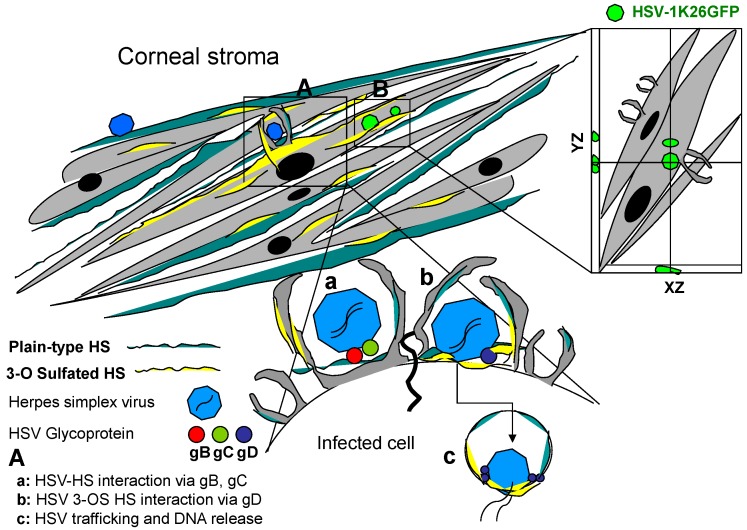
Clinical significance of heparan sulfate (HS) and 3-*O* sulfated heparan sulfate (3-*O*S HS) during herpes simplex virus (HSV) entry. Primary cultures derived from human corneal stroma: corneal fibroblast (CF) widely expresses both plain-type or unmodified HS (blue) and 3-*O*S HS (yellow). Highlighted regions in panel **A** depict the role of HS and 3-*O*S HS during HSV entry into corneal stroma. Unmodified HS expressed by CF membrane provides virus attachment or binding sites (**a**) which is mediated by two HSV glycoprotein B (gB) and C (gC). This interaction results HSV glycoprotein D (gD) to interact with the modified form of HS (3-*O*S HS) which promotes virus-cell fusion (**b**) and virus capsid trafficking via endsome (**c**) resulting fusion of capsid with endosome to release viral genome. Highlighted regions in panel **B** demonstrates the ability of HSV-1 to penetrate deep in corneal stroma via z-section of deconvolution microscopy using capsid-tagged green fluorescent virus.

In late 90s the discovery that 3-*O*ST isoform-3-generated HS allowed HSV penetration into host cells marked a landmark discovery assigning a novel structure-specific function to 3-*O*S HS [39]. The latter is generated by 3-*O*STs, which act to modify HS late in its biosynthesis [3,4,5], and each member of the 3-*O*ST family recognizes, as substrate, glucosamine residues in regions of the HS chain having specific, but different, prior modifications, including epimerization and sulfation at other positions [62,63]. Thus, each 3-*O*ST can generate potentially unique protein-binding sites within HS. To date, six different isoforms of 3-*O*STs (3-*O*ST-1, 3-*O*ST-2, 3-*O*ST-3_A_, 3-*O*ST-3_B_, 3-*O*ST-4 and 3-*O*ST-5) are known. All, except 3-*O*ST-1, generate HSV-1 entry receptors [6,63]. Interestingly, only 3-*O*ST-3_A_ and 3-*O*ST-3_B_ generate structurally identical gD receptors. The gD receptors generated by other isoforms are very similar, but likely not identical, in structure [63,64,65,66]. 3-*O*ST-1 generates binding sites for antithrombin [62,67] but fails to produce a receptor that binds to HSV-1 gD [6]. 3-*O*STs (one or more) are expressed in human and mouse tissues relevant to HSV-1 infection examined thus far [8,68,69,70,71]. Using primary cultures of CF derived from human corneal donors we provided the first clinical significance of 3-*O*S HS promoting virus entry (Figure 3) [28]. 

Since the HSV virions spread cell-to-cell *in vivo* via membrane fusion to form polykaryocytes we also provided the first visual evidence that 3-*O*S HS co-localizes with HSV-1 glycoprotein D (gD) during the membrane fusion event [42]. Interestingly, enzymatic removal of HS and 3-*O*S HS by heparinase treatment, or pre-incubation of cells with HS and 3-*O*S HS recognizing peptides significantly reduced the viral entry and spread in CF [28]. During the primary HSV-1 infection or during reactivation, the virus gets an opportunity of affecting different structural components of the cornea leading to corneal keratitis [72]. Although both the direct effects of the virus and immune mediated responses are known to cause damage to the cornea, the roles of HS and 3-*O*S HS go beyond viral entry and spread [8]. For instance, HS plays crucial role during viral attachment to the corneal epithelium, while 3-*O*S HS mediates virus-cell fusion and spread from cell to cell. Corneal infection by HSV can lead to herpetic stromal keratitis (HSK), which is a major cause for infectious blindness [73,74,75,76]. Similarly, HS is one of the major players proposed in the causation of neovascularization and angiogenesis [77,78]. They are found in free forms, in the extracellular matrix (ECM), or associated with the plasma membrane where they regulate the function of a wide range of ligands [79]. In particular, endothelial HSPGs modulate angiogenesis by affecting bioavailability and interaction of heparin-binding vascular endothelial growth factors (VEGFs) and fibroblasts growth factor (FGF) with signaling VEGFRs and their tyrosine kinase receptors [80]. Heparin/HS interaction with angiogenic growth factors depends on the degree/distribution of sulfate groups and length of the glycosaminoglycan chain, distinct oligosaccharide sequences mediating its binding activity [80,81,82]. The resulting angiogenesis compromises immune privileges of the cornea allowing extravasation of the inflammatory mediators in to the corneal stroma [72,83]. The latter event is responsible for corneal scarring and vision impairment. From cell biology standpoint the 3D constructs of corneal cells, keratocytes, exhibit long-range associations with collagen bundles in the developing matrix via an extended network of actin-rich tubular cytoplasmic protrusions-α keratopodia [84]. Interestingly, the presence of HSV not only enhances actin-rich filopodia in multiple cell types including primary cultures of corneal stroma derived from human eye donors [85] but also promotes viral spread since keratopodia are connected to adjacent cells [84]. Previous studies from our lab has shown that HSV-1 infected cell expressing 3-*O*ST-3 modified HS forms significantly higher number of filopodia than normal HS expressing cell [85,86]. Overall, HSV infection of the cornea provides a good model system to study the significance of HS and 3-*O*S HS.

## 3. Zebrafish 3-*O*ST Generated HS: A Tool to Study HSV Corneal Damage

In recent years, the zebrafish has become a favorite model organism for biologists studying infectious diseases and associated pathologies [87,88,89,90]. Included among some of the advantages are its rapid embryonic development, the transparency of its embryos for direct visual imaging of viral pathogenesis, cell and tissue specific 3-*O*ST expression in zebrafish embryo [48,91], availability of 3-*O*ST knockouts [92], and the potential for high throughput screening *in vivo* [93]. Such advantages make it an ideal model system for studying 3-*O*S HS for both basic science as well as translational aspect of the glycoscience research [87]. These characteristics are also being exploited by researchers to understand host-pathogen interaction at the level of inflammation and innate immune response to infectious disease and, accordingly, there is a growing literature on the use of zebrafish to model viral disease including the HSV infection [87].

Anatomic and ultrastructural characterization of the zebrafish cornea has demonstrated many similarities to the human cornea and providing the basis for the use of the zebrafish model both to analyze HSV spread and inflammation. At 6 months post-fertilization (mpf), the zebrafish cornea is approximately 20 μm thick and contains all five major layers found in the human cornea: the epithelium, Bowman’s layer, stroma, Descemet’s membrane, and endothelium [94]. The earlier immunostaining experiments have shown a high signal for 3-*O*ST isoform-3 in zebrafish similar to human corneal stromal fibroblasts. These results further illustrate the structural similarity between zebrafish and mammalian corneas. In addition, zebrafish corneal endothelium, like its human counterpart results in corneal edema following surgical injury and ouabain injection [94].

Further, the powerful imaging techniques and relative ease of genetic manipulation especially with the availability of 3-*O*ST isoform specific KOs have made zebrafish an attractive model system to study role of specific 3-*O*ST isoform generated HS during HSV infection of the cornea. Studying ocular infection in the intact zebrafish model is a powerful tool for several reasons. The early time point infection and associated pathologies to the other neighboring eye regions besides the infected cornea can be visualized directly in real-time and events like angiogenesis and inflammation resulting in tissue damage can be recorded. In addition, a translational aspect of using zebrafish model is the ability to screen specific 3-*O*S HS inhibitors against multiple steps from preventing viral spread, tropism to associated complications [87]. Zebrafishes’ competitive advantage over other model systems is exemplified by the optical clarity of a vertebrate embryo amenable to large-scale screenings to identify receptor-specific and small viral entry inhibitor molecules [93]. Further, toxicity of the 3-*O*S HS peptides can be studied. Therefore, the transparency of zebrafish embryos and early larvae permits observations to be made *in vivo* on intact animals, whereas similar procedures in rodents would require surgery or other invasive monitoring techniques. A further advantage of using zebrafish assays over traditional mammalian models is the short duration of such assays. For example, screens for compounds that are effective in blocking viral entry/replication/egress can be performed in intact zebrafish in one week. By comparison, in rodents, the assays for viral entry/replication will take a period between 3 and 5 weeks and the fate of the inhibitor could not be examined in the real-time.

Recently, the expression pattern of multiple isoforms of 3-*O*STs and their significance was reported in zebrafish. Cadwallader and Yost reported *in vivo* characterization of eight 3-*O*ST family members in zebrafish with seven genes showing homology to known 3-*O*ST genes in mouse and humans [91]. Interestingly, two zebrafish genes, 3-*O*ST-3X and 3-*O*ST-3Z, were found highly similar to human 3-*O*ST-3A and 3-*O*ST-3B respectively. They are likely the true homologs since their catalytic domains are near 100% identical. Such a high degree of conservation points to highly conserved functions as well. In addition, it was noted that members of zebrafish 3-*O*ST family share at least 63% similarity within the catalytic domain to the corresponding human isoform, with the exception of zebrafish 3-*O*ST-5, which showed only 53% similarity to human 3-*O*ST-5 [91]. In terms of zebrafish 3-*O*ST expression, most family members showed extensive brain expression which was restricted to very specific brain subdivisions. For instance, zebrafish 3-*O*ST-2 was expressed in developing brain, otic vesicle, and olfactory areas during early zebrafish development, while 3-*O*ST-3X was observed in neural tube and lateral plate mesoderm. Similarly, zebrafish 3-*O*ST-6 was expressed at high level in hindbrain with no expression in spinal cord region. Interestingly, structural and cell adhesion properties of zebrafish HS bearing syndecan proteins were reported to be shared with higher vertebrates [95].

The diversity in the expression of 3-*O*ST family members in a zebrafish system provided us an opportunity to examine the role of zebrafish 3-*O*ST-3 in terms of HSV-1 entry [49]. Using CHO-K1 cells that lack endogenous 3-*O*-sulfation, we demonstrated the role of zebrafish 3-*O*ST-3 in HSV-1 entry and spread. More direct and visual evidence for HSV-1 entry was demonstrated by using green fluorescent protein (GFP)-tagged HSV-1 (K26GFP) virions infecting zebrafish 3-*O*ST-3 expressing CHO-K1 cells [88]. In recent years our group has cloned and characterized all the 3-*O*ST isoforms expressed in zebrafish embryos [49,50,96,97]. Interestingly 3-*O*ST enzymes are uniquely expressed in different cells and tissues during zebrafish embryonic development [91]. To date our results with zebrafish clones to study HSV infection are very encouraging as they complement human 3-*O*ST isoform in terms of supporting HSV entry and spread [50]. The result from the study promotes the usage of zebrafish as a new model to address the role of 3-*O*ST generated HS in viral tropism, tissue specific damages in cornea and central nervous system (CNS) and associated inflammation and also for 3-*O*S HS structure-function studies for other systems. In cell culture model a unique phenotypes for HSV-1 entry was observed when individual zebrafish 3-*O*ST isoforms were tested against HSV infection. For instance one group of 3-*O*ST gene family isoforms (3-*O*ST-2, -3, -4, and -6) with conserved catalytic and substrate-binding residues of the enzyme mediated HSV-1 entry and spread, while the other group (3-*O*ST-1, -5, and -7) lacks these properties and hence did not contribute to HSV infection [50]. Taken together, our previous studies provide a clear rationale for studying cell and tissue specific HSV pathogenesis [87]. With the characterization of all other members of zebrafish 3-*O*ST for HSV infection provides unique explanation for their potential roles in HSV tropism and gD binding. For example, those zebrafish 3-*O*ST members, 3-*O*ST-3Z, 3-*O*ST-4 and 3-*O*ST-6, that are highly expressed in eye could contribute during HSV-1 infection in the eye as reported for human 3-*O*ST-3 isoform during ocular HSV infection [28]. Because zebrafish 3-*O*STs are widely expressed in brain and HSV is a neurotropic virus, they are likely to be very important for neuro-pathologies associated with HSV infection [98]. Therefore, use of zebrafish embryo model to understand *in vivo* significance of 3-*O*S HS and its interaction with gD during HSV-1 entry/spread is innovative and further enhances our ability to understand the critical regions of HS and modified HS involved in HSV pathogenesis. The current murine and rabbit models used to study HSV infection suffer from the limitation of tracking viral trafficking (entry and spread) in real-time.

The “optical clarity” in zebrafish embryos along with the expression of HS and 3-*O*S HS moieties in the zebrafish provides excellent opportunities to study real-time events during HSV entry and spread in relation to the receptor usage. Furthermore, the advantage of using a zebrafish model is to test 3-*O*ST receptor-specific inhibitors in short duration of time, which again is not possible with present murine and rabbit models against HSV infection. For instance, HSV-1 entry inhibitors generated against HS and 3-*O*ST modified HS by our group, may also turn useful to study HSV-1 induced pathological damages especially during ocular corneal infection or neuronal damages along with recurrent infections in zebrafish model because HS and 3-*O*S HS have been widely implicated for their role in assisting HSV-1 entry and spread in both ocular and neuronal cells.

## 4. Phage Display Library Screening Targeting Heterogeneous HS to Isolate Unique Peptides that Inhibit HSV Pathogenesis

Generation of peptide or mimetic targeting sulfated regions of HS offers a realistic, straightforward approach to understand HSV-3-*O*S HS interactions for novel therapeutic interventions [30]. HS in general, and with 3-*O* sulfation, aid to HSV pathogenesis at multiple steps during virus life cycle [99,100]. During initial phase of virus infections HSV glycoproteins (gB, and gC) interact with unmodified HS at cell surface or on virus activated filopodia [61]. In addition, virus binding to cell primes or activates other signaling receptors and cascades which facilitate viral entry. To promote virus-cell fusion an enzymatic modification of HS via 3-*O* sulfation generates HSV fusion receptor [6]. Similarly, endogenous HS and or 3-*O*S HS aid in virus trafficking to the nucleus [85]. It is yet to be determined whether the preferences of HSV glycoprotein for a distinct HS residue during entry, trafficking and egress differ or not? Further the pattern of sulfation in HS chain could be a molecular marker for HSV induced cell and tissue damage. Is it possible that virus controls the editing of HS modifications or locks the modifications in Gagosome?—A previously suggested place where HS biosynthetic enzymes gather and work in close proximity [101,102]. It is very clear that overall HS plays many critical roles in viral pathogenesis. Interestingly, a class of lipophilic HS mimetics has been generated that not only blocks HSV infection but also show virucidal activity against HSV-1 and HSV-2 [103,104]. This class includes PG545 [105], currently in clinical trials for cancer, which also shows potent viruclidal activity against HIV [106] and RSV [107]. In addition, a number of HS mimetic have been progressed to clinical development against cancer and proving good candidates as anti-inflammatory drugs [108,109,110,111,112,113]. The structural complexities of HS along with specific and rare sulfated modifications are the fundamental problems associated with the study of HS and modified HS. In addition due its large size, finding reagents to detect HS and/or to synthesize HS has been difficult [5]. This all has prevented the development of cell and tissue specific HS recognizing antibodies or markers for 3-*O* sulfation. One additional point of general interest is the fact that multiple pathogens including viruses use HS to initiate infection [7] and therefore, development of anti-HS and anti-3*O*S HS reagents including peptides will greatly boost biomedical research to study host-pathogen interactions and future broad spectrum drug development. Similarly, the appreciation of the structural diversity of HS species and its role in pathological conditions including viral entry and associated inflammation have been stronglyhampered by the lack of appropriate methodologies. For instance sequence strategies are not at hand, and specific antibodies, obvious tools for studying diversity, are difficult to raise. To circumvent this, we used phage display technology to map different regions of HS and 3-*O*ST generated HS to develop reagents that recognize HSV-1 gD-3-*O*S HS interaction to negatively affect HSV entry and spread [30,114].

**Figure 4:**
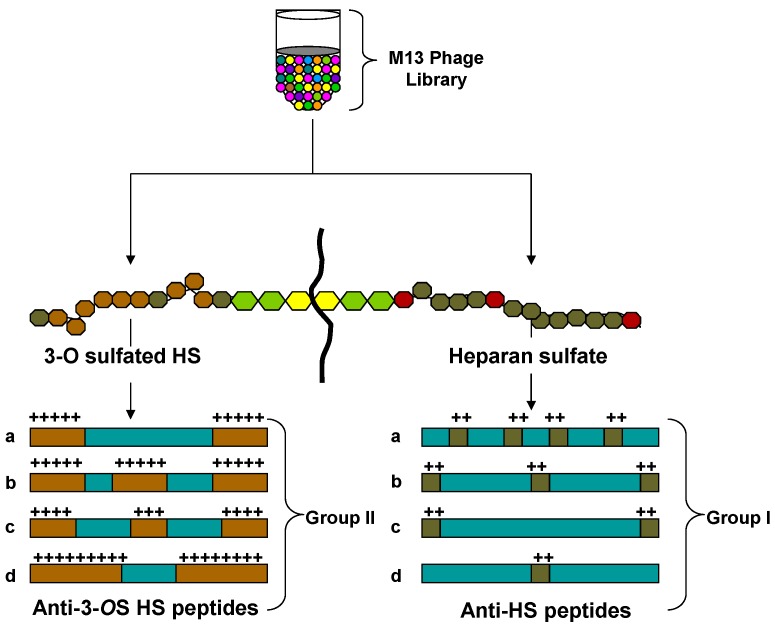
Generation of unique 12-mer peptides against diverse HS via phage display library screening. A diagrammatic presentation of random M13-phage display library screening which lead the identification of representative candidates from two-different groups of anti-HS peptides with high positive charge densities. Group I, represented by G1 peptide (LRSRTKIIRIRH), belongs to a class with alternating charges (XRXRXKXXRXRX), and group II, represented by G2 peptide (MPRRRRIRRRQK), shows repetitive charges (XXRRRRXRRRXK).

Multiple rounds of screening of phages from a 12-mer peptide phage display library that targeted HS and 3-*O*S HS resulted in the enrichment of phages that bound HS or 3-*O*S HS [30]. By determining the nucleotide sequences of the portion of the phage genome that encoded the peptide sequences from individual plaques were used to determine peptide sequences. The predicted peptide sequences of about 200 plaques were determined and sorted into two groups on the basis of their targets. A frequently repeating peptide sequence from each group was subsequently selected for further characterization. As previously reported the two most frequently isolated peptide sequences LRSRTKIIRIRH (called G1 for HS binding group 1) and MPRRRRIRRRQK (G2 for 3-*O*S HS binding group 2) were synthesized and examined for their abilities to inhibit HSV entry. Anti-HS G1 peptide probably recognizes HS moieties that may not be 3-*O* sulfated. Anti-3-*O*S HS G2 peptide, on the other hand, recognizes HS moieties with 3-*O* sulfation present. Among the structural differences between G1 and G2 peptide, it appears that G2 peptide showed more dependence on the positively charged residues than G1, which is probably also dependent on the presence of a Lys residue at the N terminus. In general, arginine has been found important for charge-charge interaction with HS [30] (Figure 4). Interestingly both the peptides were able to block HSV-1 entry into CHO-K1 cells expressing one of the three gD receptors, 3-*O*S HS, nectin-1, and herpesvirus entry mediator protein (HVEM). In addition using mouse corneal model of HSV infection we further demonstrated the efficacy of the peptides in blocking infection *in vivo*, but it also shows that HS is an important HSV-1 coreceptor, not only for cultured cells but also for the cells *in vivo* [30].

In addition, we also tested G2 peptide against HSV-2 infection in a mouse model [114].Our animal study also provided a first-time proof of the importance of blocking of 3-*O*S HS during HSV-2 infection *in vivo* [30,114]. In addition our group and others have previously shown that octasacchride generated through chemo enzymatic synthesis, dendrimers based molecule targeting HS also inhibit HSV infection [34,36,115]. Additional reports further expand the critical role of sulfated HS in sexually transmitted diseases such as HIV, hepatitis B virus (HBV) and HPV [7]. A recent study demonstrated a higher affinity for CD4—an entry receptor for HIV by conjugating to a HS-mimetic peptide [116,117]. Interestingly in context of HSV a unique possibility of cell and tissue tropism exists based on population of 3-*O*S HS expressed on a given cell and tissue. For instance, 3-*O*ST-2 and 4 isoforms are highly expressed in brain tissue compared to 3-*O*ST-3 isoform which is expressed in the corneal stroma [28,41].

The major potential for such cell and tissue specific 3-*O*ST expression can be used to develop novel inhibitors which target HSV tropism. To date 3-*O*ST isoform specific HS chains have not been fully mapped against HSV gB and gD. In addition regions or domains of sulfated HS generated by multiple *O* sulfations and their implication on signaling and pathologies (angiogenesis and leukocytes migrations) are also lacking in HSV models. Interestingly our previous data indicates that HSV infection up-regulates the expression of HS carrying transmembrane protein syndecans [118,119].

## 5. Anti-HS and Anti-3-*O*S HS Peptide: A Novel Tool to Study Virus Associated Inflammation

**Figure 5:**
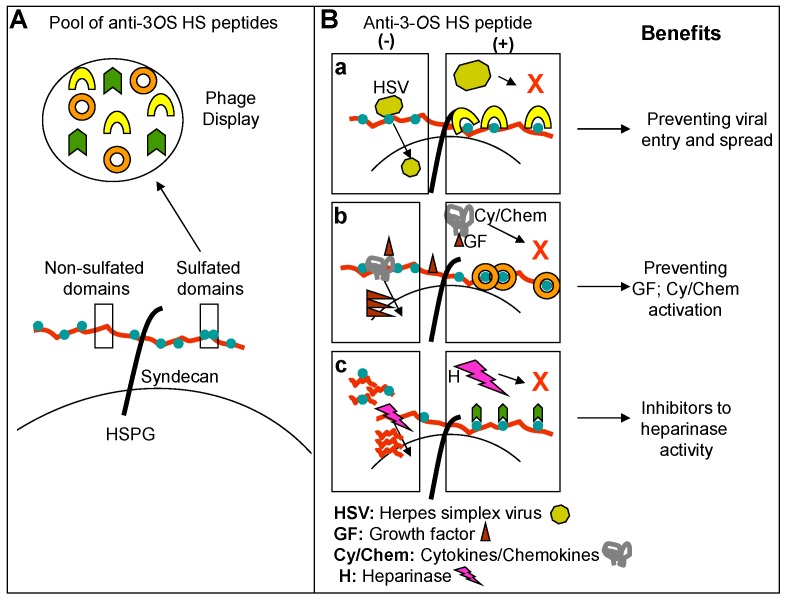
Potential use of anti-3-*O*S HS peptide generated against 3-*O*S HS. Panel **A** demonstrate isolation of wide range of anti-3-*O*S HS peptides screening against diverse 3-*O*S HS as a target. Panel **B** suggests the benefits of the isolated anti-3-*O*S HS peptides. Situations in presence (+) and absence (−) of anti-3-*O*S HS peptides are presented. In panel (**a)**, blocking of HSV-1 gD interaction to 3-*O*S HS would likely to develop viral entry inhibitors. Similarly inhibiting interaction between 3-*O*S HS and growth factors (GF)/Cytokine (Cy)/Chemokines (Chem) (**b**) and preventing heparinase activity via anti-3-*O*S HS peptides (**c**) would like to likely to develop anti-inflammatory drug candidates.

Diverse groups of HS binding protein include cytokines/cheomokines which interact with unique set of “saccharide sequences” in HS chain to recruit inflammatory cells [120,121,122] therefore preventing those critical interactions is a novel strategy to control inflammation (Figure 5). 

During HS-mediated recruitment of leucocytes at the site of inflammation or injury, HSPG regulates the gradients of chemokines and cytokines which are stimulated in response to tumor necrosis factor alpha (TNFα)—a pro-inflammatory factor. A number of chemokines and cytokines have been implicated during this processes, which include interleukin family (IL-2, -3, -4, -5, -7, -8, -10 and -12), granulocytes macrophages colony-stimulating factor (GM-CSF), regulated on activation, normal T cell expressed and secreted (RANTES), and monocyte chemotactic protein (MCP-1 and MCP-4) [121,122]. The binding of IL-8 to cell surface HSPG is highly significant in recruiting neutrophils to inflammatory sites [123]. Interestingly patient corneas with HSK have been shown to express high levels of GM-CSF and IL-8 [124]. Therefore many of these molecules represent a promising therapeutic target during neutrophil-mediated tissue destruction. Several line of evidence indicates that HS interactions to chemokines not only protect them from proteolysis but enhance their chemokine activity via oligomerization. In addition, HS aids in immobilization of chemokines on the surface of endothelial cells- an event that leads leukocytes migration to blood vessels. Further, the enhanced expression of heparinase enzyme results in the release of critical sequences of HS for tissue remodeling and angiogenesis during chronic inflammatory response.

Several HS-binding growth factors (FGF-2 and VEGEF) are known to participate in angiogenesis. In fact HS-binding polypeptides are already implicated as potential anti-angiogenic drug in cancer therapy [10,77,125,126]. Interestingly it was discovered that knocking down of one of the HS modifying enzyme, called 6-*O*-sulfotransferase, in zebrafish with morpholino antisense oligonucleotides reduced vascular branching and corresponded to changes in the HS structure [127]. It has been suggested that both oligosaccharides and small molecule biosynthetic enzyme inhibitors could be valuable HS-based strategies for controlling aberrant angiogenesis in diseases as diverse as cancer and heart disease. In this regard peptides that compete for HS and 3-*O*S HS regions required for sequestering chemokines, cytokines and growth factors will be useful for studying inflammation (Figure 5). In addition, whether HSV entry blocking peptide would also interfere HSPG mediated inflammation needs to be investigated. Similarly evaluating the anti-angiogenic potential of anti-3-*O*S HS peptides in the mouse cornea model will also be advantageous as high vasculature activities in the cornea lead to severe scarring and blindness during HSK. Despite of current understanding on the role of HS and 3-*O*S HS in HSV entry, inflammation and angiogenesis, many questions still needs to be addressed. For instance, the precise regulation of 3-*O*ST enzymes or other enzymes (2-*O* and 6-*O* sulfotransferases) involved in HS modifications in healthy cornea *vs.* HSV infected and inflamed cornea remains poorly understood. Further, which HSV glycoprotein or combination of glycoproteins and the counter sequences in HS impact the corneal tissue remodeling and angiogenesis? Similarly the other aspects like role of 3-*O*S HS dependent signaling in leukocytes recruitment, extravasations and migration, and release of cytokine and chemokines and activation of innate immune cells need to be investigated in context to the corneal damage. Modifications of HS by 3-*O*STs have generated interest in the field of viral entry. As our understanding of 3-*O*S HS is expanding on its role in inflammation and angiogenesis; in the future we will be able to rationally design 3-*O*S HS based therapeutics to prevent viral infection and associated cell and tissue damages. These strategies will need additional workout in defining 3-*O*ST expression levels in the cornea during HSK, clinical isolates of HSV and their dependence on 3-*O*S HS, and the regions of the targets involved in cell and tissue remodeling during pathogenesis. Designing novel drugs that target multiple events during HSV corneal pathogenesis are encouraged. The goal is to prevent both virus spread as well as long term chronic inflammation. The availability of 3-*O*ST isoform specific zebrafish KO embryos is a valuable tool for investigating the role of 3-*O* sulfation during HSV induced corneal damage. The applicability of anti-3-*O*S HS peptides as drugs has been suggested against HSV ocular infection [30]; however, novel small molecule mimetics may provide a better alternative with high degree of specificity. For instance, a study has shown that HS-mimetic PI-88 targets HSV-2 via gD [128]. At the current time, only a few such molecules have been identified with virtually no anti-herpes activities demonstrated *in vivo*. Therefore, more work is needed before their promise as anti-herpes drug can be proven. 

## 6. Conclusions

Molecular diversity in the HS chain is remarkable as it generates extraordinary binding sites for multiple protein ligands [1,2,3,4]. One such example is the sulfation at the C3 position of glucosamine residues in HS chain via 3-*O*ST enzymes [5], which results a unique receptor for HSV entry and cell-to-cell spread [6]. Using phage display mapping we have established the proof-of-concept that 3-*O*S HS plays a significant role during HSV infection [30]. Since the glycobiology-virology based information on sulfated HS is constantly evolving [129,130,131], therefore, the precise synthesis of HS mimetic with required charges, and relative positions of the sulfate groups will likely aid in designing potent anti-inflammatory molecules [10,77,125,126]. Such candidates will in turn advance the development of HS based therapeutics to control HSV induced corneal scarring and blindness and may offer help to rationalize prevention strategy against multiple other viruses dependent to sulfated HS [6,132]. 
